# Broad-spectrum antiviral potential of vitexin and isovitexin from *Jatropha integerrima*: in vitro cytoprotective effects and in silico insights

**DOI:** 10.1007/s00210-026-05186-z

**Published:** 2026-04-02

**Authors:** Hala Sh. Mohammed, Shimaa M. Khalifa, Eman F. S. Taha, Amal H. Ahmed, Ibrahim H. Eissa, Ahmed M. Metwaly, Mohamed Marzouk

**Affiliations:** 1 Department of Pharmacognosy and Medicinal Plants, Faculty of Pharmacy (Girls), Al-Azhar University, Cairo, 11754 Egypt; 2 Department of Health Radiation Research, National Centre for Radiation Research and Technology, Egyptian Atomic Energy Authority (EAEA), Cairo, Egypt; 3 Department of Pharmaceutical Medicinal Chemistry & Drug Design, Faculty of Pharmacy (Boys), Al-Azhar University, Cairo, 11884 Egypt; 4 Department of Pharmacognosy & Medicinal Plants, Faculty of Pharmacy (Boys), Al-Azhar University, Cairo, 11884 Egypt; 5 Department of Tanning Materials and Leather Technology, Chemical Industries Research Institute, National Research Centre, 33 El-Bohouth St. (Former El-Tahrir St.), Dokki, Cairo, 12622 Egypt

**Keywords:** Vitexin, Isovitexin, *Jatropha integerrima*, Antiviral activity, Molecular docking

## Abstract

The ongoing emergence of viral infections underscores the need for new antiviral agents with broad-spectrum potential, particularly those derived from natural products. In this study, leaf and flower extracts of *Jatropha integerrima*, along with two isolated flavone C-glycosides, vitexin, and isovitexin, were evaluated for their antiviral potential against a panel of human DNA and RNA viruses, including hepatitis A virus, herpes simplex virus types 1 and 2, adenovirus, Coxsackievirus B4, and rotavirus A. Cytotoxicity and virus-induced cytopathic effects were assessed in Vero cells using an MTT-based assay, allowing estimation of 50% cytotoxic concentrations (CC₅₀), 50% inhibitory concentrations (IC₅₀), and selectivity indices (SI). The extracts and isolated compounds exhibited variable degrees of cytoprotective effects in infected cells, with vitexin and isovitexin showing favorable selectivity profiles against selected viruses. Morphological observations supported the MTT findings, indicating partial preservation of cell integrity under certain treatment conditions. To explore potential molecular interactions relevant to coronavirus infection, in silico molecular docking was performed against five SARS-CoV-2–related targets, including Mpro, spike glycoprotein, nucleocapsid phosphoprotein, the ACE2 receptor–RBD complex, and nsp10. Both flavonoids generated favorable binding poses within the defined binding regions of the selected proteins, with predicted binding energies suggesting potential structural compatibility with these targets. These computational findings are presented as hypothesis-generating insights and do not constitute experimental evidence of antiviral efficacy against SARS-CoV-2. Overall, the findings suggest that vitexin and isovitexin possess in vitro cytoprotective effects in virus-infected cells and exhibit predicted interactions with viral targets in silico. However, given the indirect nature of the antiviral assays and the absence of direct viral replication measurements, the results should be interpreted cautiously. Further studies employing direct virological endpoints are required to validate the antiviral potential of these compounds and to clarify their mechanisms of action.

**Graphical Abstract:**

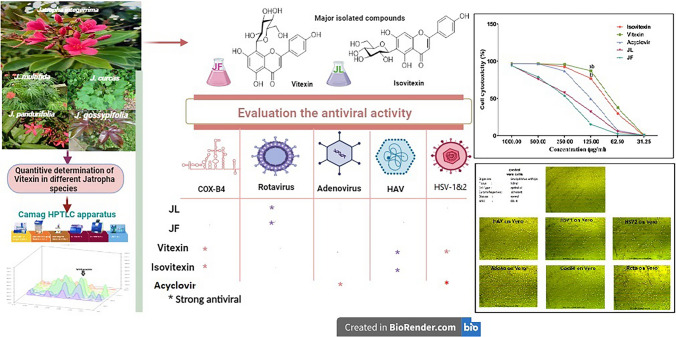

**Supplementary Information:**

The online version contains supplementary material available at 10.1007/s00210-026-05186-z.

## Introduction

Viral infections remain a major global health challenge, driven by the continual emergence of new viral pathogens and the re-emergence of established viruses with limited therapeutic options (Abdelrahim et al. 2023). Despite the availability of several antiviral drugs, their clinical utility is often constrained by narrow activity spectra, drug resistance, adverse effects, and high development costs (Abdelsayed 2025). These limitations highlight the ongoing need for novel antiviral agents with improved safety profiles and broader mechanisms of action.

Natural products have historically played a pivotal role in antiviral drug discovery, serving as direct therapeutic agents or as lead structures for synthetic optimization (Alfano et al. 2020). Among these, plant-derived flavonoids have attracted considerable attention due to their structural diversity and broad range of biological activities, including antioxidant, anti-inflammatory, and antiviral effects (Al-Khayri et al. 2022). Several flavonoids have been reported to interfere with viral entry, replication, and assembly, either through direct interactions with viral proteins or by modulating host-cell pathways essential for viral propagation (Babakhani et al. 2025).

The recent coronavirus disease 2019 (COVID-19) pandemic has further intensified interest in natural compounds with potential antiviral and immunomodulatory properties. In addition to respiratory manifestations, SARS-CoV-2 infection has been associated with dysregulated immune responses and inflammatory complications (Chagas et al. 2022). Rare but severe conditions, such as hemophagocytic lymphohistiocytosis and Sweet syndrome, have been reported following SARS-CoV-2 infection or vaccination, highlighting the complex interplay between viral infection, immune activation, and inflammatory signaling pathways (Chua 2020).

In this context, recent clinical reports provide further evidence of immune-mediated complications. (Zhang et al. 2023) described cases of hemophagocytic lymphohistiocytosis following COVID-19 vaccination, emphasizing excessive cytokine activation and systemic inflammation (Chung 2025). Similarly, (Jia et al. 2023) documented vaccine-associated Sweet syndrome, a neutrophilic dermatosis reflecting acute inflammatory responses. Collectively, these findings underscore the clinical relevance of immune dysregulation in COVID-19–related settings and support continued exploration of antiviral candidates with additional cytoprotective or immunomodulatory properties (De Clercq 2006).

The genus Jatropha (Euphorbiaceae) comprises numerous species traditionally used in folk medicine for the treatment of infectious and inflammatory disorders. Phytochemical investigations of Jatropha species have revealed a diverse array of bioactive constituents, including diterpenes, lignans, and flavonoids (Félix-Silva et al. 2018). *Jatropha integerrima *has been reported to possess antimicrobial, antioxidant, and anti-inflammatory activities (Hassan et al. 2015); however, systematic evaluation of its antiviral potential and identification of its active antiviral constituents remains limited.

Vitexin (apigenin-8-C-β-D-glucopyranoside) and isovitexin (apigenin-6-C-β-D-glucopyranoside) are naturally occurring flavone C-glycosides widely distributed in medicinal plants. These compounds have been associated with diverse pharmacological effects, including antioxidant, anti-inflammatory, and antiviral activities against selected viral pathogens (Hernández et al. 2025). Their chemical stability and favorable safety profiles make them attractive candidates for further antiviral evaluation. Nevertheless, comparative assessments of their antiviral potential across multiple virus families, as well as mechanistic insights into their possible interactions with viral targets, remain insufficiently explored.

In vitro antiviral screening commonly employs cytopathic effect-based assays to evaluate the capacity of compounds to protect host cells from virus-induced damage (Idrees et al. 2024). Although such assays provide useful preliminary insights, they offer indirect evidence of antiviral activity and may be influenced by compound-related cytotoxicity or host-cell modulation (Islam et al. 2026). Accordingly, careful interpretation and transparent reporting of assay limitations are essential to ensure accurate assessment of antiviral potential.

In parallel with experimental screening, in silico molecular docking has emerged as a valuable tool for exploring potential interactions between small molecules and viral protein targets (Jia et al. 2023; Kaba 2025). While docking studies cannot substitute for experimental validation, they may offer hypothesis-generating insights into possible binding modes and guide future mechanistic or structure-based investigations (Khan et al. 2021). In the context of SARS-CoV-2, several viral proteins involved in replication and host interaction have been widely investigated as potential therapeutic targets using computational approaches (Hierholzer 1996).

Against this background, the present study aimed to evaluate the antiviral potential of leaf and flower extracts of *Jatropha integerrima* and the isolated flavone C-glycosides vitexin and isovitexin against a panel of human DNA and RNA viruses using in vitro cytopathic effect-based assays. In addition, in silico molecular docking was employed to explore the potential interactions of vitexin and isovitexin with selected SARS-CoV-2 protein targets. By integrating experimental cytoprotective assessments with computational analyses, this work seeks to provide a balanced and cautious evaluation of the antiviral potential of these compounds while clearly acknowledging the limitations inherent to the applied methodologies.

## Materials and methods

### Plant material and identification

Fresh leaves and flowers of *Jatropha integerrima* were collected in June 2018 from El-Orman Botanical Garden, Giza, Egypt (30°01′44.5″N, 31°12′46.7″E). Botanical identification was performed and authenticated by Dr. Tearse Labib (Department of Flora and Taxonomy, El-Orman Botanical Garden). A voucher specimen (No. 14) was deposited in the Herbarium of the Department of Pharmacognosy and Medicinal Plants, Faculty of Pharmacy, Al-Azhar University, Cairo, Egypt. The scientific name was verified using The Plant List database.

### Reagents and chemicals

All solvents and reagents were of analytical grade. Methanol, ethyl acetate, formic acid, petroleum ether, and distilled water were purchased from Merck (Darmstadt, Germany). Pre-coated silica gel 60 F254 HPTLC plates (20 × 10 cm) were obtained from Merck. Acyclovir was purchased from Sigma-Aldrich (St. Louis, MO, USA).

### Extraction and isolation of flavone C-glycosides

Air-dried powdered leaves and flowers (2 kg each) of *J. integerrima* were extracted with 70% aqueous methanol (8 × 3 L) at 70 °C. The combined extracts were concentrated under reduced pressure using a rotary evaporator (Büchi, Switzerland). The residues were defatted with petroleum ether and subsequently dissolved in methanol to eliminate sugars and inorganic constituents. The methanol-soluble leaf fraction (198 g) was subjected to column chromatography over polyamide (S6) and eluted using a stepwise H₂O/MeOH gradient. Elution was performed sequentially with distilled water, followed by increasing concentrations of methanol (10%, 20%, 40%, 60%, and 100% MeOH), using the indicated volumes for each step. Fractions were monitored by paper chromatography under UV light and visualized using appropriate spray reagents. Fractions III (4.3 g) and IV (1 g), which exhibited similar chromatographic profiles, were combined and further purified on a cellulose column using 20–40% aqueous ethanol. Subsequent purification was achieved by repeated chromatography on Sephadex LH-20 using n-butanol:acetic acid:water (4:1:5, v/v/v), followed by methanol. This procedure yielded vitexin (1.8 g; purity > 98% by HPLC) and isovitexin (32 mg; purity > 96% by HPLC). Structural elucidation was performed using ^1^H and ^13^C NMR spectroscopy and mass spectrometry. Representative spectral data were provided in the Supplementary Material (Figures S1–S6).

### HPTLC fingerprinting and quantification

HPTLC analysis was performed using a CAMAG system equipped with a Linomat 5 applicator, TLC Scanner 3, and winCATS software (version 1.3.4). Chromatographic development was carried out in a saturated chamber (20-min saturation) using a mobile phase of ethyl acetate:methanol:water:formic acid (50:2:3:6, v/v/v/v). Plant extracts were prepared at a concentration of 20 µg/mL in methanol. A vitexin standard stock solution (1000 µg/mL) was prepared in methanol, and working standards ranging from 2.5 to 20 µg/mL were applied in 5 µL volumes. Quantification was based on the linear regression equation (y = 13256x + 485.7; *R*^2^ = 0.9987), where y represents peak area and x represents concentration (µg/mL). Linearity was established over the range of 2.5–20 µg/mL.

Method validation demonstrated limits of detection (LOD) and quantification (LOQ) of 0.78 µg/mL and 2.36 µg/mL, respectively. Intra-day and inter-day precision, % RSD values were below 2% at concentrations of 5, 10, and 15 µg/mL. Recovery values ranged from 97.8% to 101.2%. Method robustness was evaluated by introducing minor variations in mobile phase composition, chamber saturation time, and detection wavelength, with % RSD remaining below 2%. Vitexin content in plant extracts was calculated using the validated calibration curve.

### Cell culture and virus propagation

Vero cells (African green monkey kidney, CCL-81; ATCC, USA) were cultured in Dulbecco’s Modified Eagle Medium (DMEM) supplemented with 10% fetal calf serum, 2 mM L-glutamine, 100 U/mL penicillin, and 100 µg/mL streptomycin. Cells were maintained at 37 °C in a humidified incubator containing 5% CO₂ (Kumari et al. 2025). The viruses used in this study included hepatitis A virus (HAV), herpes simplex virus type 1 (HSV-1), herpes simplex virus type 2 (HSV-2), adenovirus type 40, Coxsackievirus B4 (CoxB4), and rotavirus A (SA11 strain). Viral stocks were propagated in confluent Vero cell monolayers and harvested upon reaching 90–100% cytopathic effect (CPE). Virus suspensions were clarified and stored at − 80 °C until use (Latif and Nawaz 2025).

### Cytotoxicity assay

Cytotoxicity was evaluated using the MTT assay. Vero cells were seeded in 96-well plates at a density of 1 × 10^4^cells per well and allowed to adhere overnight. Serial dilutions of extracts and isolated compounds (maximum concentration 1000 µg/mL) were prepared in DMEM supplemented with 10% fetal calf serum. Vehicle (DMSO) and untreated controls were included. After 24 h of treatment, 20 µL of MTT solution (5 mg/mL in PBS) was added to each well and incubated for 4–5 h. The resulting formazan crystals were dissolved in 200 µL DMSO, and absorbance was measured at 570 nm using a microplate reader (Ma et al. 2021) Cell viability and cytotoxicity percentages were calculated as described in Section "Calculation of cytotoxicity and antiviral activity" (Magnavacca et al. 2022). The 50% cytotoxic concentration (CC₅₀) was determined by nonlinear regression analysis using GraphPad Prism v9.5.1. The maximum non-toxic concentration (MNTC) was defined as the highest concentration that did not significantly reduce cell viability compared with untreated control cells (*p*< 0.05) (Mahrous et al. 2026).

### Antiviral assays

Antiviral activity was assessed using a cytopathic effect-based MTT assay. Vero cells were seeded at 1 × 10^4^cells per well and infected with virus at multiplicities of infection (MOI) of 0.1 for HAV, adenovirus, and rotavirus A, and 0.01 for HSV-1, HSV-2, and CoxB4. Following 1 h of viral adsorption, inocula were removed, cells were washed with PBS, and fresh medium containing 2% fetal calf serum and non-cytotoxic concentrations of the test compounds was added (Markova et al. 2025; Marzouk et al. 2023).

Infected cells were incubated for 24 h post-infection before MTT assessment To ensure accurate interpretation of antiviral activity, the following control groups were included in each experiment:

- Uninfected untreated control (cell control): Vero cells without viral infection or treatment, representing 100% cell viability.
- Infected untreated control (virus control): Vero cells infected with virus but receiving no treatment, representing maximal virus-induced cytopathic effect.
- Vehicle control: Vero cells treated with the highest concentration of DMSO used in the test samples (final concentration ≤ 0.1%, v/v) to exclude solvent-related effects.
- Cytotoxicity control: Uninfected cells treated with the corresponding concentration of each test compound to confirm that observed effects were not due to compound-induced cytotoxicity.
- Positive control: Acyclovir was used as a reference antiviral drug only for HSV-1 and HSV-2 infections, due to its established anti-herpetic activity. No mechanistically specific reference drug was included for HAV, adenovirus, CoxB4, or rotavirus A (Meager 2004; Mediani et al. 2025).

The 50% inhibitory concentration (IC₅₀) was calculated from dose–response curves using nonlinear regression analysis. The selectivity index (SI) was calculated as CC₅₀/IC₅₀. All experiments were performed in three independent biological replicates conducted on different days, each including technical triplicates.

For the mixed treatment assay, virus suspensions were pre-incubated with test compounds at their respective MNTCs (1:1, v/v) for 1 h at room temperature before being added to Vero monolayers (Nagarajan 2022). After 24-h incubation, cell viability was determined by MTT assay as described above (Naithani et al. 2008).

### Calculation of cytotoxicity and antiviral activity

The percentages of cell viability, cytotoxicity, and antiviral activity were calculated from MTT absorbance values measured at 570 nm using the following formulas:

Percentage of cell viability:

$$\%\;Cell\;Viability=\left(\frac{Abs_{treated}}{Abs_{control}}\right)\times100$$

where *Abs treated* represents the absorbance of treated cells and *Abs control* represents the absorbance of untreated control cells.

Percentage of cytotoxicity:

$$\%\;Cytotoxicity=100-\%\;Cell\;Viability$$

For antiviral evaluation, the percentage of antiviral activity was calculated as following:

$$\%\,Antiviral\;Activity=\left(\frac{Abs_{treated\;infected}-Abs_{infected\;control}}{Abs_{uninfected\;control}-Abs_{infected\;control}}\right)\times100$$

where.

- *Abs treated infected* = absorbance of infected cells treated with the tested sample
- *Abs infected control* = absorbance of infected untreated cells
- *Abs uninfected control* = absorbance of uninfected untreated cells

The selectivity index (SI) was calculated as following:

$$SI=\frac{C{C}_{50}}{I{C}_{50}}$$

where CC₅₀ represents the 50% cytotoxic concentration and IC₅₀ represents the 50% inhibitory concentration.

### In silico molecular docking

Molecular docking studies were conducted using MOE 2014.0 (Chemical Computing Group, Montreal, Canada). Three-dimensional structures of SARS-CoV-2 protein targets, including main protease (PDB ID: 6LU7), spike glycoprotein (6VYB), nucleocapsid protein (6VYO), ACE2 receptor complexed with the spike receptor-binding domain (PDB ID: 6M17), and nonstructural protein 10 (6W4H), were retrieved from the Protein Data Bank.

Protein structures were prepared by removing water molecules and co-crystallized ligands, adding hydrogen atoms, assigning protonation states at pH 7.4, and performing energy minimization using the AMBER99 force field until the root-mean-square gradient reached 0.01 kcal/mol/Å. Binding sites were defined based on co-crystallized ligands or reported active-site residues. Grid box dimensions were adjusted to encompass the entire active site region.

Vitexin and isovitexin structures were constructed, protonated at physiological pH, and energy minimized using the MMFF94x force field. Docking was performed using the Triangle Matcher placement method, with London dG scoring for initial ranking. The top poses were refined using an induced-fit protocol and rescored using the GBVI/WSA dG scoring function. Thirty poses were generated per ligand per target. Docking scores were used for comparative analysis only and do not represent experimental binding free energies.

Docking protocol validation was performed by redocking co-crystallized ligands into their original binding sites. Root-mean-square deviation (RMSD) values below 2.0 Å were considered indicative of reliable docking performance (Naithani et al. 2008; Organization 2024).

### Statistical analysis

All experiments were performed as three independent biological replicates conducted on separate days, with each condition tested in technical triplicate within each experiment. Data were presented as mean ± standard error (SE). Statistical analyses were carried out using GraphPad Prism version 9.5.1 (GraphPad Software, San Diego, CA, USA). Differences among groups were analyzed using one-way analysis of variance (ANOVA), followed by Tukey’s or Bonferroni’s post hoc test as appropriate. A *p*-value < 0.05 was considered statistically significant. The 50% cytotoxic concentration (CC₅₀) and 50% inhibitory concentration (IC₅₀) values were determined by nonlinear regression analysis using a log(inhibitor) versus normalized response model with variable slope.

## Results

### Identification of isolated compounds

**Fig. 1:**
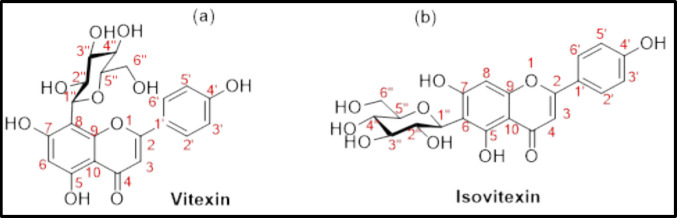
Chemical structures of vitexin (**a**) and isovitexin (**b**)

Vitexin and isovitexin were successfully extracted and recognized from the leaves of *J. integerrima* (Fig. 1a and b). The identification of these isolated compounds was confirmed by High-Resolution Electrospray Ionization Mass Spectrometry (HRESI-MS), Proton Nuclear Magnetic Resonance (^1^H NMR), and Carbon-13 Nuclear Magnetic Resonance (^13^C NMR). The chemical structures of vitexin and isovitexin were confirmed by NMR and MS analyses (Figures S1–S6).

Apigenin 8-C-β-D-glucopyranoside (Vitexin, Fig. 1a): The compound was a pale yellow, amorphous powder. It exhibits *R*_*f*_ values of 0.41 (BAW) and 0.16 (15% AC) on paper chromatography. Under UV light, a dark purple spot transitions to a greenish-yellow color in the presence of NA/PE. The application of FeCl_3_ reagents results in a green color. Upon complete acid hydrolysis, there is no observable change.

(˗)-HRESI-MS (Figure S1): *m/z* 431.0972 [M‒H]^‒^, 341.0660 [M‒H‒90]^‒^, and 311.0566 [M‒H‒120]^‒^.^1^H NMR (400.19 MHz, DMSO-*d*_*6*_, Figure S2): δ ppm 13.18 (1H, brs, OH-5), 10.84, 10.35 (each 1H,brs, 2 × aromatic OH), 8.03 (2H, d, *J* = 8.4 Hz, H-2‵/6‵), 6.89 (2H, d, *J* = 8.4 Hz, H-3‵/5‵), 6.79 (1H, s, H-3), 6.28 (1H, s, H-6), 4.99, 4.71 (each 1H,brs, 2 × aliphatic OHs), 4.69 (1H, d, *J* = 9.6 Hz, H-1‶ hidden by OH), 3.80 (2H, m, H-2‶ and H-6‶a), 3.60–3.20 (4H, m, hidden by H_2_O-signal, H-3‶, 4‶, 5‶, 6‶b). ^13^C NMR (100.63 MHz, DMSO-*d*_*6*_, Figure S3): δ ppm 182.6 (C-4), 164.4 (C-2), 163.0 (C-7), 161.6 (C-4‵), 160.9 (C-5),156.5 (C-9), 129.4 (C-2‵/6‵), 122.1 (C-1‵), 116.3 (C-3‵/5‵), 105.1 (C-8), 104.5 (C-10), 102.9 (C-3), 98.6 (C-6), 82.3 (C-5‶), 79.1 (C-3‶), 73.8 (C-1‶), 71.3 (C-2‶), 71.0 (C-4‶), 61.8 (C-6‶).

Apigenin 6-*C*-β-D-glucopyranoside (Isovitexin, Fig. 1b): The compound is a pale yellow amorphous powder. On paper chromatography, it exhibits *R*_*f*_ values of 0.46 (BAW) and 0.18 (15% AC). Under UV light, a dark purple spot transitions to a greenish-yellow color when exposed to NA/PE. The application of FeCl_3_ reagents results in a green color. Notably, there is no observable change upon complete acid hydrolysis.

(˗)- HRESI-MS (Figure S4): *m/z* 431.2294 [M‒H]^‒^. ^1^H NMR (850.15 MHz, DMSO-*d*_*6*_, Figure S5): δ ppm 13.56 (1H, brs, OH-5), 7.93 (2H, d, *J* = 8.5 Hz, H-2‵/6‵), 6.93 (2H, d, *J* = 8.5 Hz, H-3‵/5‵), 6.79 (1H, s, H-3), 6.51 (1H, s, H-8), 4.58 (1H, d, *J* = 9.4 Hz, H-1‶), 4.04 (H-2‶, t-like, *J* = 8.5 Hz), 3.80 (brd, H-6‶a), 3.60–3.10 (4H, m, hidden by H_2_O-signal, H-3‶, H-4‶, H-5‶, H-6‶b). ^13^C NMR (213.79 MHz, DMSO-*d*_*6*_, Figure S6): *δ* ppm 182.4 (C-4), 164.0 (C-2), 163.8 (C-7), 161.7 (C-4‵), 161.1 (C-5), 156.7 (C-9), 128.9 (C-2‵/6‵), 121.6 (C-1‵), 116.5 (C-3‵/5‵), 109.4 (C-6), 103.8 (C-10), 103.2 (C-3), 94.1 (C-8), 82.1 (C-5‶), 79.4 (C-3‶), 73.5 (C-1‶), 71.1 (C-2‶), 70.07 (C-4‶), 61.9 (C-6‶).

### Antiviral assay results

#### Cytotoxicity and determination of maximum non-toxic concentration (MNTC)

**Fig. 2:**
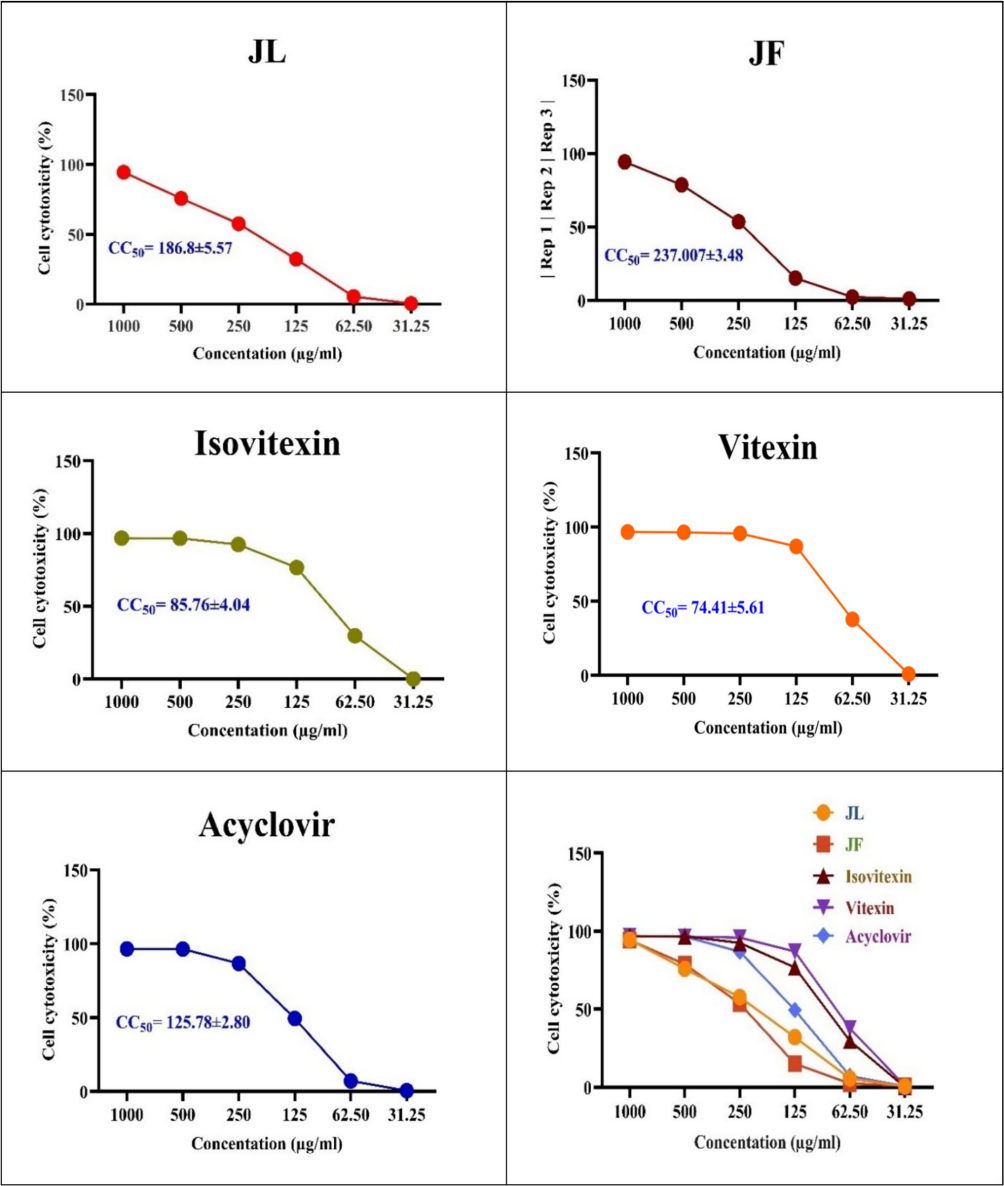
Dose-dependent cytotoxicity of JL, JF, isovitexin, vitexin, and acyclovir in Vero cells determined by the MTT assay. Data represent mean ± SE (*n* = 3)

The cytotoxicity of *J. integerrima* leaf (JL) and flower (JF) extracts, together with the isolated flavone C-glycosides isovitexin and vitexin, was evaluated in Vero cells using the MTT assay prior to antiviral assessment. Determination of CC₅₀ and MNTC values ensured that subsequent antiviral effects were not attributable to nonspecific cytotoxicity (Table 1, Fig. 2). All tested samples exhibited concentration-dependent cytotoxicity. Morphological alterations, including reduced confluence and cellular detachment, were observed at concentrations between 31.25 and 1000 µg/mL. The CC₅₀ values were 186.80 ± 5.57 µg/mL for JL, 237.00 ± 3.48 µg/mL for JF, 85.76 ± 4.04 µg/mL for isovitexin, and 74.41 ± 5.61 µg/mL for vitexin. Acyclovir showed a CC₅₀ of 125.78 ± 2.80 µg/mL. Statistical analysis revealed no significant difference between the CC₅₀ values of vitexin and isovitexin (*p* > 0.05), indicating comparable cytotoxic profiles.

**Table 1:** Cytotoxicity of *J. integerrima* Jacq. leaf (JL) and flower (JF) extracts, isovitexin, and vitexin compared to acyclovir at various concentrations on the Vero cell line using the MTT assay

ID	µg/mL	Viability %	Toxicity %	CC_50_ µg/mL	MNTC µg/mL
**Vero**	–––	100	0		
**JL**	1000	5.60	94.39	186.80 ± 5.57^ac^	62.5
	500	24.15	75.84		
	250	42.30	57.69		
	125	67.88	32.11		
	62.5	94.49	5.50		
	31.25	99.59	0.40		
**JF**	1000	5.81	94.18	237.00 ± 3.48^ab^	62.5
	500	21.20	78.79		
	250	46.48	53.51		
	125	84.91	15.08		
	62.5	97.65	2.34		
	31.25	98.98	1.01		
**Isovitexin**	1000	3.16	96.83	85.76 ± 4.04^abc^	31.25
	500	3.28	96.71		
	250	7.52	92.47		
	125	23.33	76.66		
	62.5	70.27	29.72		
	31.25	99.93	0.063		
**Vitexin**	1000	3.22	96.77	74.41 ± 5.61^abc^	31.25
	500	3.54	96.45		
	250	4.23	95.76		
	125	13.02	86.97		
	62.5	62.30	37.69		
	31.25	99.24	0.75		
**Acyclovir**	1000	3.41	96.58	125.78 ± 2.80^bc^	62.5
	500	3.60	96.39		
	250	13.21	86.78		
	125	50.53	49.46		
	62.5	92.78	7.21		
	31.25	99.49	0.50		

Data were expressed as mean ± SE (*n* = 3). CC_50_, 50% cytotoxic concentration; MNTC, maximum non-toxic concentration. Statistical analysis was performed using one-way ANOVA followed by Tukey’s post hoc test. ^a^Significant related to acyclovir, ^b^significant related to JL, and csignificant related to JF at *p* < 0.05

The MNTC was determined as 62.5 µg/mL for JL and JF extracts and 31.25 µg/mL for both isolated flavones, consistent with the MNTC of acyclovir. Among all tested samples, JF exhibited the highest CC₅₀ value, indicating the widest safety margin in Vero cells. These MNTC values were used in all subsequent antiviral experiments.

#### Dose–response cytotoxicity profile

Dose–response analysis confirmed a progressive reduction in cell viability with increasing concentrations (Fig. 2). At MNTC levels, all samples maintained cell viability above 95%, confirming minimal cytotoxicity under experimental conditions. The crude extracts demonstrated lower cytotoxicity compared to the isolated flavones. At 125 µg/mL, vitexin exhibited significantly higher cytotoxicity than JL (*p* < 0.05) and JF (*p* < 0.01). Similarly, isovitexin showed significantly greater cytotoxicity than JF at the same concentration (*p* < 0.05). These findings indicate a broader safety margin for the crude extracts relative to the purified compounds.

#### Effects on cellular morphology of Vero cells

**Fig. 3:**
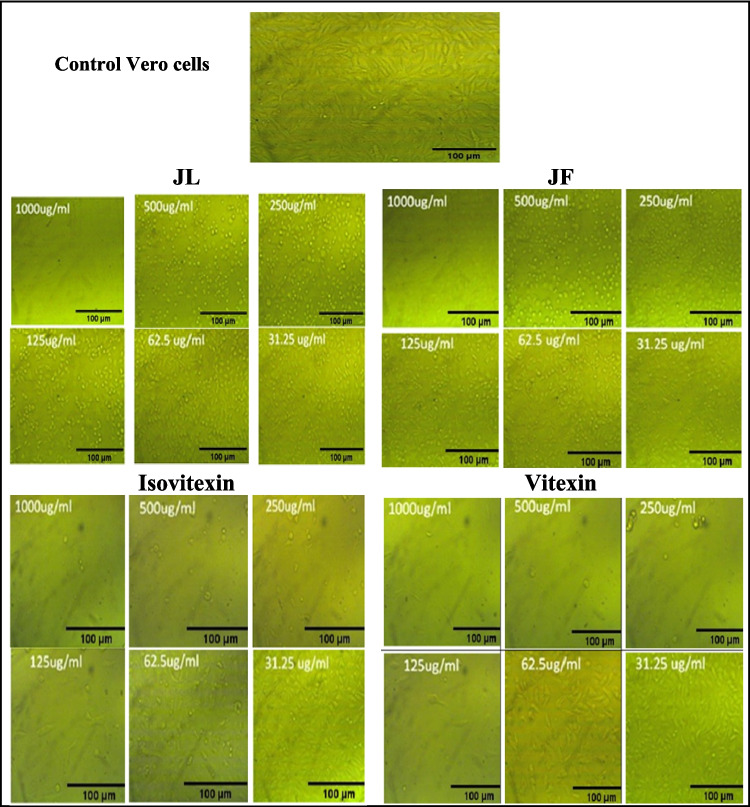
Representative micrographs showing morphological changes in Vero cells after 7 days of treatment with increasing concentrations of JL, JF, isovitexin, and vitexin compared with untreated control cells (100 × magnification), scale bar (100 µm)

Microscopic evaluation over a 7-day period revealed dose-dependent morphological changes (Fig. 3). At higher concentrations, treated cells exhibited reduced density, loss of spindle-shaped morphology, cellular shrinkage, cytoplasmic granulation, and detachment from the culture surface. These alterations were consistent with the cytotoxicity data obtained from the MTT assay. At the established MNTCs, Vero cells maintained a confluent monolayer with morphology comparable to untreated controls, with no observable signs of toxicity. These observations confirm that the selected MNTCs represent safe concentrations for antiviral evaluation.

#### Cell viability at MNTC

**Fig. 4:**
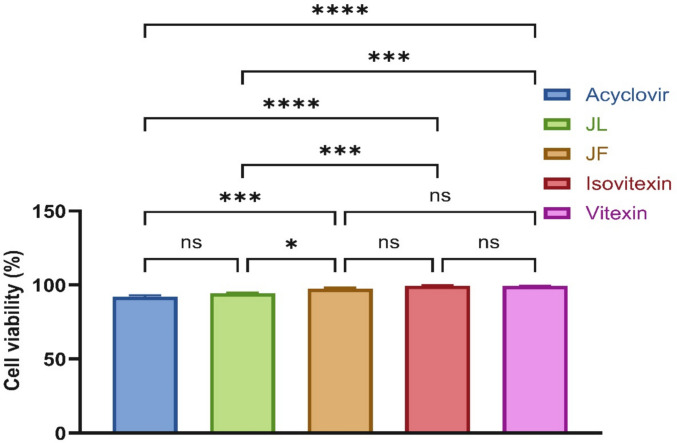
Cell viability (%) of Vero cells at the respective MNTC of each tested sample. Data were presented as mean ± SE (*n* = 3). A one-way ANOVA represented the data, Tukey post hoc test at *p* < 0.05

At their respective MNTCs, all tested samples demonstrated minimal cytotoxicity. The percentage reduction in cell viability was 0.06% for isovitexin, 0.75% for vitexin, 5.50% for JL, and 2.34% for JF. In comparison, acyclovir induced 7.21% cytotoxicity at its MNTC (Fig. 4). Among the tested samples, isovitexin exhibited the lowest cytotoxic effect at its MNTC. These results confirm that the selected concentrations were appropriate for antiviral screening and that observed antiviral activity is unlikely to result from nonspecific cytotoxicity.

#### Virus-induced cytopathic effects (CPE)

**Fig. 5:**
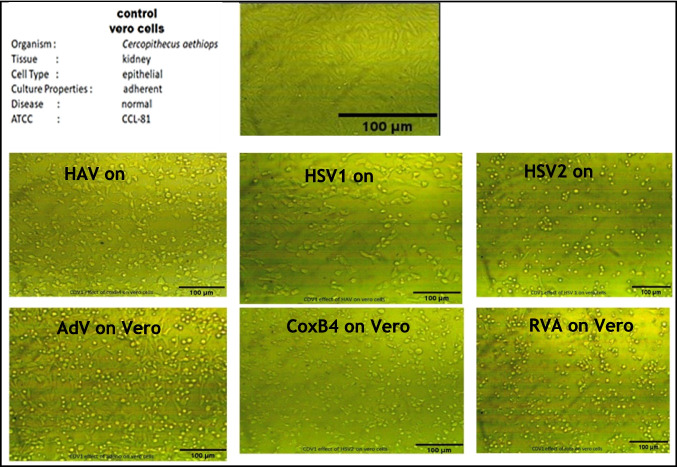
Microscopic morphology of Vero cells before and after infection with HAV, HSV-1, HSV-2, AdV, CoxB4, and RVA. Post-infection cells exhibited distinct cytopathic effects (CPE), including partial and complete loss of the monolayer, cell rounding, shrinkage, and granulation, alongside cytoplasmic protrusions and vacuolization (100 × magnification), scale bar (100 µm)

Infection of Vero cells with HAV, HSV-1, HSV-2, AdV, CoxB4, and RVA resulted in distinct virus-associated cytopathic effects (Fig. 5). Observed morphological alterations included nuclear condensation, cell rounding, cytoplasmic blebbing, vacuolization, and partial or complete disruption of the monolayer. The HSV-1 induced the most severe cytopathic damage, characterized by extensive aggregation of non-viable cells and marked monolayer destruction. CoxB4 also produced pronounced morphological alterations. In contrast, RVA caused comparatively moderate cytopathic changes with smaller cellular aggregates. The remaining viruses produced measurable but variable degrees of cellular injury. These findings confirm successful viral infection and establish a morphological basis for subsequent antiviral evaluation.

### Viral cytopathogenicity on Vero cell line

**Fig. 6:**
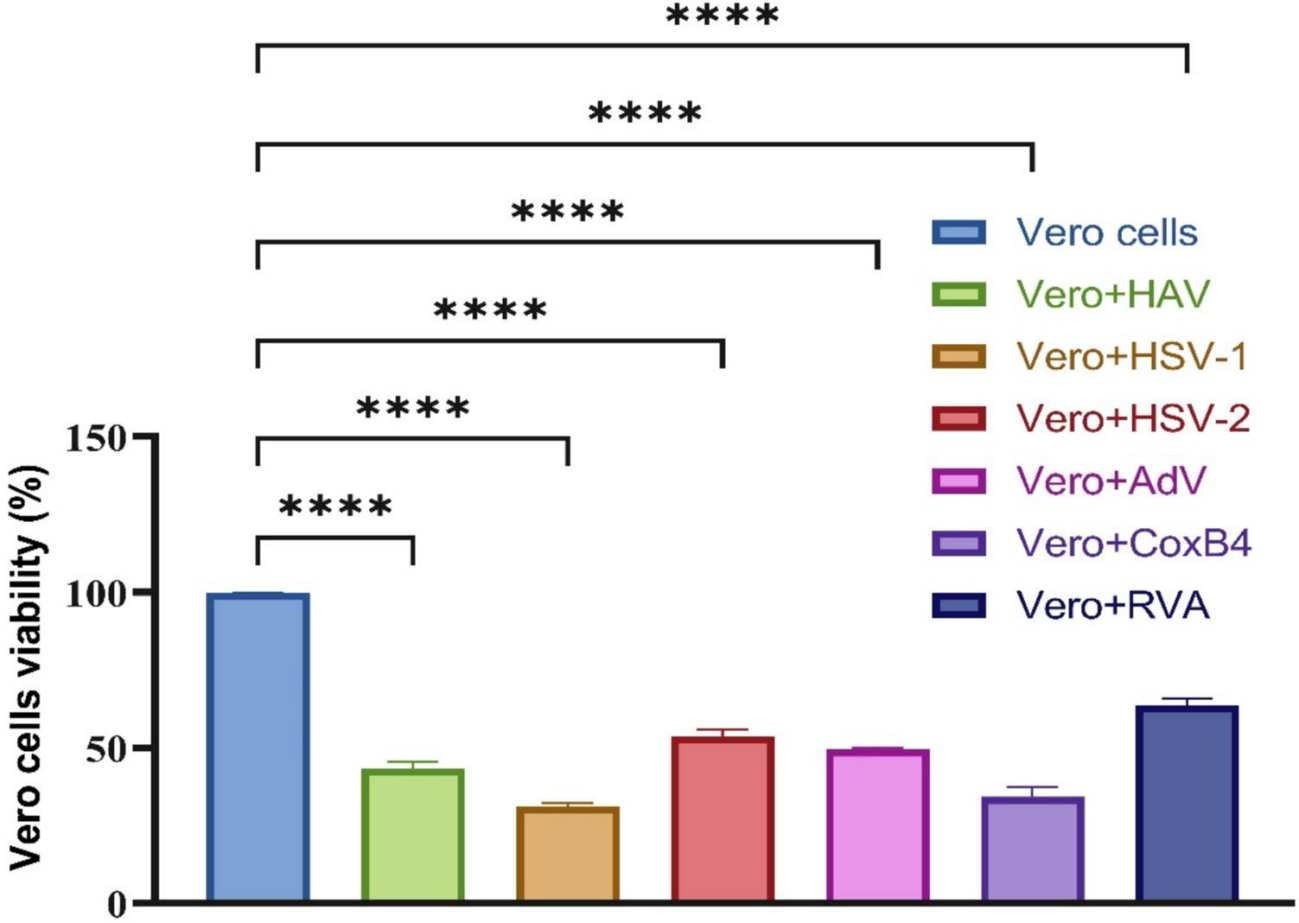
Impact of viral infection on Vero cell viability. Data represent mean ± SE of three independent experiments (*n* = 3). A significant difference compared with uninfected control Vero cells (one-way ANOVA followed by Tukey’s post hoc test, *p* < 0.05)

To quantitatively assess virus-induced cellular damage, Vero cell viability was measured relative to uninfected controls (Fig. 6). Infection with all tested viruses resulted in a statistically significant reduction in cell viability (*p* < 0.05), confirming effective induction of cytopathic effects.

HSV-1 and CoxB4 produced the most pronounced reductions in viability, whereas HAV, AdV, and HSV-2 caused moderate decreases. RVA exhibited the least cytopathogenic effect among the tested viruses. These quantitative findings support the reproducibility and suitability of the infection model for antiviral assessment.

### In vitro antiviral activity

The antiviral activity of JL, JF, isovitexin, and vitexin was evaluated at their respective maximum non-toxic concentrations (MNTCs) against the tested viral panel. Treatment with the tested samples significantly improved cell viability compared with infected untreated controls (Table 2). Against HAV, the isolated flavone C-glycosides demonstrated marked antiviral efficacy, with vitexin and isovitexin restoring cell viability to near-control levels. In HSV-1 infection, vitexin and isovitexin exhibited strong antiviral activity, with effects comparable to acyclovir. Similarly, in HSV-2 infection, vitexin demonstrated antiviral activity approaching that of acyclovir. For AdV and CoxB4, the flavone C-glycosides exhibited variable antiviral responses, with vitexin showing the highest activity among the tested plant-derived compounds against CoxB4. Against RVA, the crude extracts, particularly JF, demonstrated relatively higher antiviral activity compared with the isolated flavones. Overall, the flavone C-glycosides exhibited notable antiviral activity across several tested viruses, with particularly strong activity against HSV-1 and HSV-2.

**Table 2:** Antiviral efficacy (%) of *J. integerrima* extracts, isovitexin, vitexin, and acyclovir against a panel of human viruses at their respective MNTC

Tested materials					
Viruses	**JL**	**JF**	**Isovitexin**	**Vitexin**	**Acyclovir**
HAV	74.26 ± 3.4^cde^	90.44 ± 0.6^ade^	97.61 ± 2.5^ae^	98.87 ± 4.1^ae^	
HSV-1	53.39 ± 5.8^bcde^	32.06 ± 2.0^acde^	78.57 ± 2.8^ab^	85.57 ± 2.2^ab^	83.16 ± 6.7^ab^
HSV-2	46.65 ± 2.1^cde^	38.97 ± 3.7^cde^	59.50 ± 5.7^abd^	74.38 ± 6.5^abc^	70.46 ± 4.3^ab^
AdV	4.76 ± 4.7^cde^	5.15 ± 2.0^cde^	15.10 ± 3.4^abde^	29.12 ± 2.5^abce^	
CoxB4	59.08 ± 4.3^bcde^	71.19 ± 4.3^acd^	88.16 ± 4.5^abe^	98.06 ± 4.3^abe^	
RVA	20.34 ± 7.2^c^	30.97 ± 0.8^ce^	8.14 ± 3.8^abd^	20.74 ± 3.8^c^	

Data represent the mean percentage of antiviral activity ± SE (*n* = 3). Superscript letters (a–e) indicated statistically significant differences (*p* < 0.05) based on one-way ANOVA followed by Tukey’s post hoc test: Superscript letters (a–d) indicate statistically significant differences (p < 0.05) relative to JL, JF, isovitexin, and vitexin, respectively. For HSV-1 and HSV-2 only, comparisons with acyclovir were performed

#### Dose–response analysis and IC₅₀ determination

**Fig. 7:**
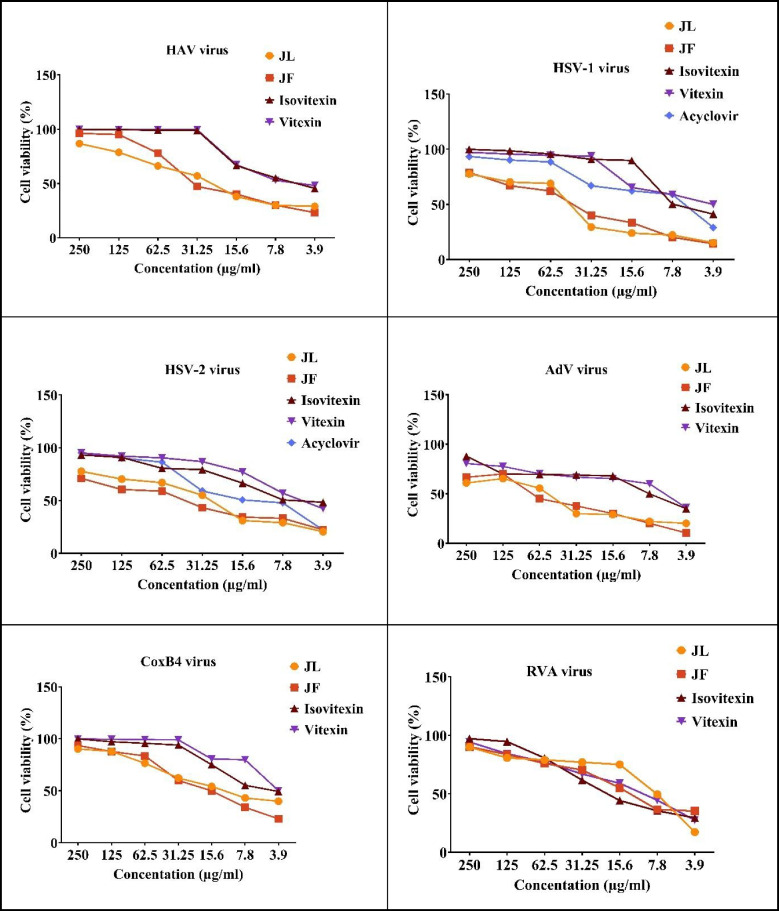
Dose–response curves illustrating the inhibitory concentration (IC_50_) and the antiviral effect of *J. integerrima* extracts, isovitexin, vitexin, and acyclovir against a panel of human viruses (HAV, HSV-1, HSV-2, AdV, CoxB4, and RVA) using the MTT assay. Acyclovir was used as a reference for DNA viruses (HSV-1 and HSV-2) only; its activity against RNA viruses is not representative

Dose–response curves were generated for JL, JF, isovitexin, vitexin, and acyclovir against the viral panel using the MTT assay. The concentrations required to inhibit virus-induced cytopathic effects by 50% (IC₅₀) were calculated from nonlinear regression analysis (Fig. 7). These IC₅₀ values were subsequently used to determine selectivity indices. It should be noted that acyclovir was included as a reference compound for HSV-1 and HSV-2 (DNA viruses) and may not provide a mechanistically relevant comparison for RNA viruses such as HAV and CoxB4.

### Selectivity index (SI) and pharmacological potency

The selectivity index (SI), calculated as CC₅₀/IC₅₀, was used to evaluate therapeutic potential (Table 3). Higher SI values indicate greater antiviral selectivity. Vitexin and isovitexin exhibited the highest SI values against CoxB4 and HSV-1 among the tested plant-derived compounds. Against HAV, both flavones showed favorable selectivity indices compared with the crude extracts. In HSV-1 and HSV-2 infections, the SI values of vitexin and isovitexin were comparable to that of acyclovir. For AdV and RVA, selectivity varied among samples, with crude extracts demonstrating relatively higher SI values against RVA. These findings indicate that vitexin and isovitexin possess promising selectivity profiles, particularly against HSV-1 and HSV-2.

**Table 3:** Cytotoxicity (CC_50_), inhibitory concentration (_IC50_), and selectivity index (SI) of tested samples against the viral panel

Test materials						
**Viruses**		**JL**	**JF**	**Isovitexin**	**Vitexin**	**Acyclovir**
	**CC**_**50**_** (µg/mL)**	186.80 ± 5.57	237.00 ± 3.48	85.76 ± 4.04	74.41 ± 5.61	125.78 ± 2.80
**HAV**	IC_50_ (µg/mL)	25.24 ± 3.20	28.56 ± 1.28	5.63 ± 0.49	5.04 ± 1.73	
	SI	7.40	8.30	15.23	14.76	
**HSV-1**	IC_50_ (µg/mL)	48.55 ± 1.13	39.02 ± 1.38	5.36 ± 1.09	3.95 ± 0.63	9.40 ± 1.1
	SI	3.85	6.07	16.00	18.83	13.38
**HSV-2**	IC_50_ (µg/mL)	27.88 ± 1.13	40.65 ± 1.15	6.58 ± 1.15	6.42 ± 1.19	14.44 ± 1.32
	SI	6.70	5.83	13.03	11.59	8.71
**AdV**	IC_50_ (µg/mL)	64.16 ± 1.27	62.75 ± 1.84	8.93 ± 1.15	7.20 ± 1.22	
	SI	2.91	3.78	9.60	10.33	
**CoxB4**	IC_50_ (µg/mL)	17.20 ± 1.20	16.40 ± 1.27	4.13 ± 1.25	3.90 ± 1.11	
	SI	10.86	14.45	20.77	19.08	
**RVA**	IC_50_ (µg/mL)	10.90 ± 1.18	13.50 ± 1.16	20.66 ± 1.12	13.70 ± 1.27	
	SI	17.14	17.56	4.15	5.43	

*CC₅₀*, 50% cytotoxic concentration (µg/mL); *IC₅₀*, 50% inhibitory concentration (µg/mL); *SI*, selectivity index (CC₅₀/IC₅₀). Values are mean ± SE (*n* = 3). Higher SI values indicate greater antiviral selectivity

### In silico molecular docking analysis against SARS-CoV-2 targets

Molecular docking simulations were performed to evaluate the potential interactions of vitexin and isovitexin with key SARS-CoV-2 proteins, including the main protease (Mpro), spike glycoprotein, nucleocapsid phosphoprotein, membrane glycoprotein, and nonstructural protein 10 (nsp10) (Table 4).

**Table 4:** Predicted binding energies (*ΔG*, kcal/mol) of vitexin and isovitexin against selected SARS-CoV-2 protein targets

Protein target	PDB ID	Co-crystallized ligand	*ΔG* Ligand (kcal/mol)	*ΔG* Vitexin (kcal/mol)	*ΔG* Isovitexin (kcal/mol)	RMSD (Å)
Main protease Mpro	6LU7	PRD_002214	− 8.23	− **6.12**	− 5.80	1.42
Spike glycoprotein	6VYB	NAG	− 3.56	− 4.96	− **5.10**	1.68
Nucleocapsid	6VYO	MES	− 3.80	− 5.19	− **5.35**	1.51
ACE2 receptor–RBD complex	6M17	NAG	− 3.63	− 4.88	− **5.05**	1.73
Nonstructural protein 10 (nsp10)	6W4H	SAM	− 6.08	− **6.48**	− 6.20	1.39

*ΔG* values represent predicted binding energies (kcal/mol). RMSD values (< 2.0 Å) were considered indicative of acceptable reproduction of native binding poses only for targets containing authentic co-crystallized ligands occupying defined binding sites (i.e., Mpro and nsp10). For structures containing crystallization additives such as NAG or MES, RMSD values were not regarded as formal validation metrics. The lowest Δ*G* between vitexin and isovitexin for each target is highlighted in bold. NAG (N-acetyl-D-glucosamine) represents glycosylation residues present in the crystal structure and does not constitute a pharmacological inhibitor. MES is a crystallization buffer component and was not considered a functional co-crystallized nhibitor

Both flavone C-glycosides showed predicted binding affinities toward the examined targets. Vitexin exhibited its strongest predicted affinity toward nsp10 (− 6.48 kcal/mol), while isovitexin showed comparable binding to the same target (− 6.20 kcal/mol). For spike, nucleocapsid, ACE2–RBD complex, and nsp10 proteins, in some cases, the calculated docking scores were comparable to those of the co-crystallized ligands; however, docking scores represent relative estimates and do not imply superior biological activity. Redocking validation was performed only for targets containing authentic co-crystallized ligands occupying defined binding sites (e.g., Mpro and nsp10). For these targets, RMSD values below 2.0 Å indicated acceptable reproduction of the native binding pose. For structures lacking pharmacologically relevant co-crystallized inhibitors (e.g., NAG or MES), RMSD values were calculated for docking pose consistency but were not considered formal validation of the docking protocol. Overall, the docking results indicate that both compounds were capable of interacting with multiple viral targets, suggesting the possibility of multi-target binding interactions at the computational level. However, these findings were derived from computational modeling and should be interpreted as predictive rather than confirmatory evidence of antiviral activity.

#### Detailed binding mode analysis

**Fig. 8:**
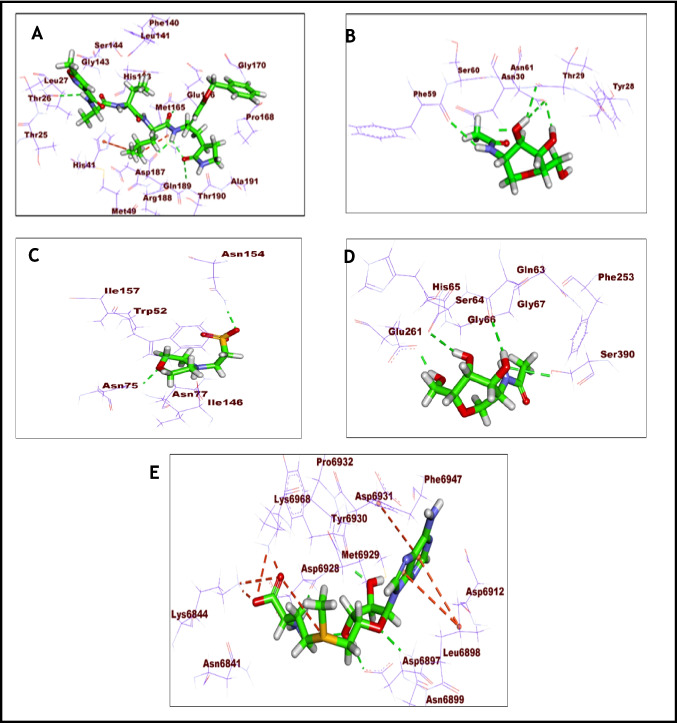
Binding mode of the co-crystallized ligand docked into the active site of SARS-CoV-2 targets: **A** main protease; **B** spike glycoprotein; **C** nucleocapsid phosphoprotein; **D** ACE2 receptor–RBD complex; and **E** Nsp10

**Fig. 9:**
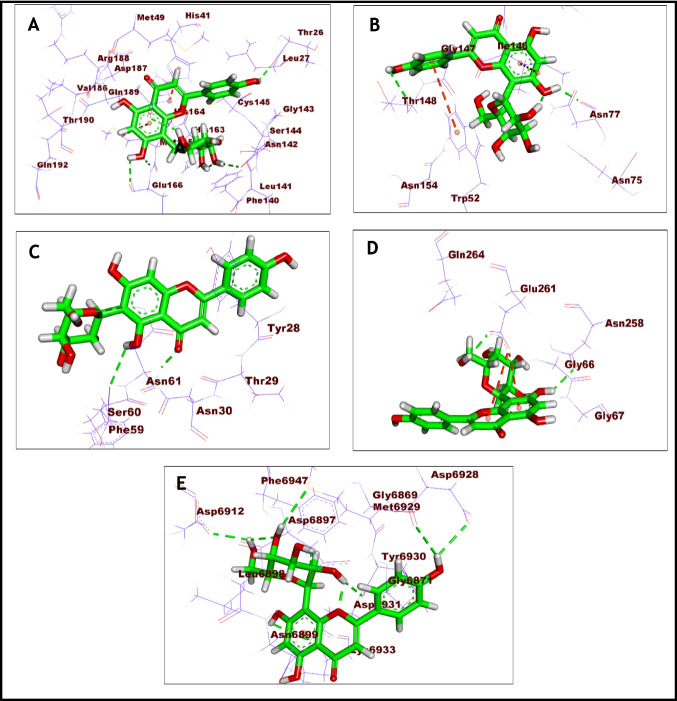
Docking pose of the vitexin ligand within the active site of SARS-CoV-2 targets: **A** main protease; **B** spike glycoprotein; **C** nucleocapsid phosphoprotein; **D** ACE2 receptor–RBD complex; and **E** Nsp10

**Fig. 10:**
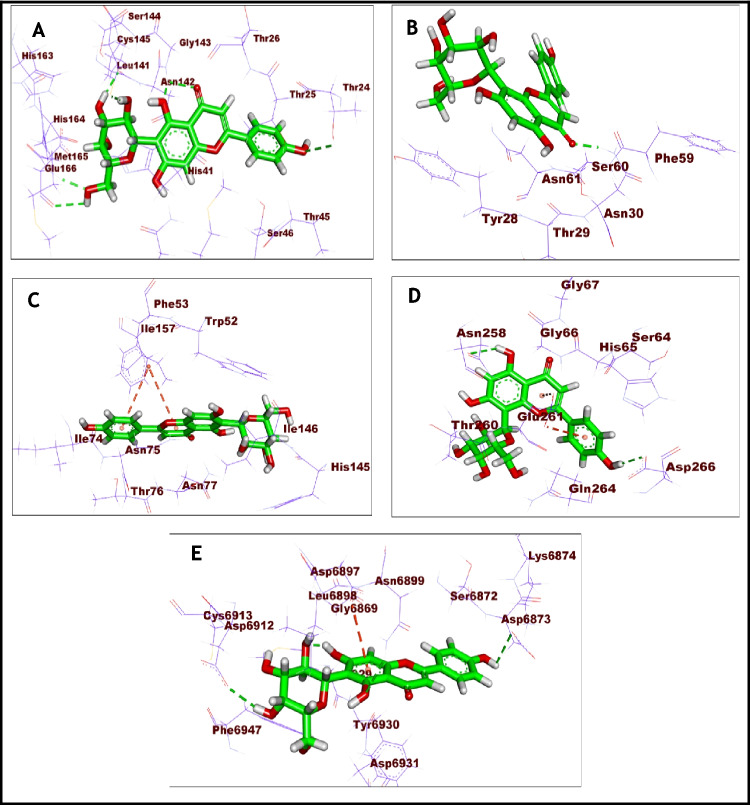
Docking pose of the isovitexin ligand within the active site of SARS-CoV-2 targets: **A** main protease; **B** spike glycoprotein; **C** nucleocapsid phosphoprotein; **D** ACE2 receptor–RBD complex; and **E** Nsp10

Detailed interaction analysis was conducted to characterize the binding orientations and intermolecular contacts of vitexin and isovitexin within the active sites of the selected SARS-CoV-2 proteins (Figs. 8, 9, and 10).

Within the Mpro active site, vitexin formed six hydrogen bonds involving His163, Glu166, Thr26, Asn142, and Gln189, along with hydrophobic interactions with His41 and Met165. Isovitexin established seven hydrogen bonds with Thr24, Gly143, Glu166, Leu141, and Cys145, suggesting favorable accommodation within the catalytic cleft based on docking prediction.

In the spike glycoprotein binding region, vitexin formed a hydrogen bond with Asn30, whereas isovitexin formed three hydrogen bonds with Asn30, Asn61, and Phe59, consistent with its slightly more favorable binding energy for this target.

For the nucleocapsid phosphoprotein, the human ACE2 receptor in complex with the spike receptor-binding domain (ACE2–RBD complex), and nsp10, both compounds established multiple hydrogen bonds within the predicted binding pockets. Notably, vitexin formed seven hydrogen bonds in the nsp10 complex, corresponding to its lowest calculated binding energy among the evaluated targets.

These interaction patterns support the possibility that vitexin and isovitexin may bind stably within functional regions of multiple viral proteins. Nevertheless, the docking data represent theoretical predictions and require experimental validation to confirm biological relevance.

### HPTLC quantification

Vitexin content was qualitatively and quantitatively assessed in methanolic extracts of leaves and stems from selected Jatropha species using the validated HPTLC method described above.

Chromatographic separation was performed on silica gel 60 F254 plates using a mobile phase of ethyl acetate:methanol:water:formic acid (50:2:3:6, v/v/v/v), following a 20-min chamber saturation period. Vitexin was well resolved at an *R*_*f*_ value of 0.53. A vitexin stock solution (1000 µg/mL) was prepared in methanol, and calibration standards ranging from 2.5 to 20 µg/mL were applied. Dried plant materials were subjected to ultrasonic-assisted extraction, and resulting extracts (20 µg/mL) were analyzed under identical chromatographic conditions.

**Fig. 11:**
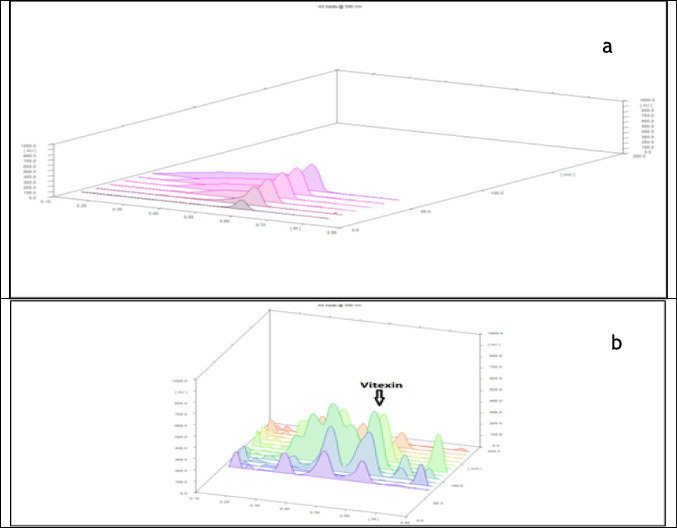
**a** 3D display of all tracks of vitexin standards at 340 nm. **b** 3D display of all tracks of the examined plants at 340 nm

Vitexin identification was confirmed by matching Rf values and UV spectra of sample peaks with those of the isolated reference compound, as illustrated in Fig. 11a and b.

**Fig. 12:**
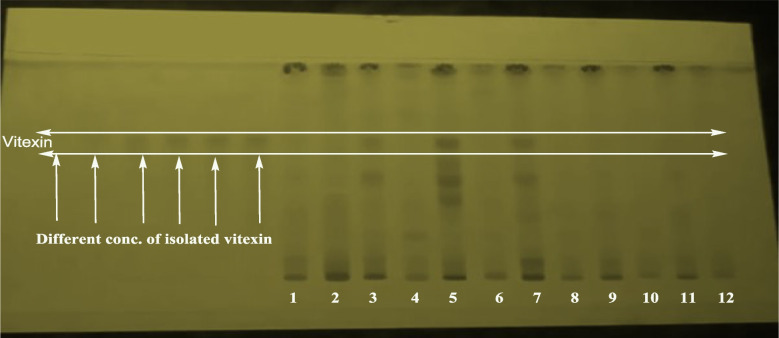
Developed HPTLC plate showing vitexin standard and extracts of selected Jatropha species. Tracks correspond to leaf and stem extracts as indicated

Quantitative analysis demonstrated that leaf extracts consistently contained higher vitexin concentrations than corresponding stem extracts. Among the examined species, Jatropha gossypifolia, Jatropha multifida, and Jatropha curcas leaves exhibited the highest vitexin content. As summarized in Table 5, Jatropha gossypifolia leaves showed the highest vitexin concentration (0.352%, equivalent to 352.266 µg/g dry weight), whereas *Jatropha integerrima* leaves contained 0.038% (38.105 µg/g dry weight). The developed HPTLC chromatogram is presented in Fig. 12.

**Table 5:** Vitexin concentration in the investigated plant samples

No	Plant	Part	Vitexin (% w/w)	No	Plant	Part	Vitexin (% w/w)
1	*J. integerrima* Jacq	Leaves	0.0381	7	*J. curcas* L	Leaves	0.2236
2	*J. integerrima* Jacq	Stems	0.0003	8	*J. curcas* L	Stems	0.0001
3	*J. multifida* L	Leaves	0.2559	9	*J. pandurifolia* Andrews	Leaves	0.0818
4	*J. multifida* L	Stems	0.0004	10	*J. pandurifolia* Andrews	Stems	0.0032
5	*J. gossypifolia* L	Leaves	0.3522	11	*J. integerrima* Rosea	Leaves	0.0298
6	*J. gossypifolia* L	Stems	0.0035	12	*J. integerrima* Rosea	Stems	0.0034

## Discussions

Current antiviral therapies, most of which are chemically synthesized, are frequently limited by adverse effects, narrow spectra of activity, and the emergence of drug-resistant viral strains (Ouedraogo et al. 2025). These challenges highlight the need for alternative antiviral agents with improved safety profiles and broader mechanisms of action. Natural products remain an important source of bioactive compounds for infectious disease management (Ouedraogo et al. n.d)). Among these, flavonoids have attracted particular attention due to their documented antiviral, antioxidant, and anti-inflammatory properties (Raj and Varadwaj 2016; Reddy et al. 2023).

In addition to direct viral cytopathogenicity, immune-mediated pathology has emerged as a major determinant of disease severity in viral infections, including COVID-19. Clinical reports have described rare but severe inflammatory complications following SARS-CoV-2 infection or vaccination. For example, (Zhang et al. 2023) reported cases of hemophagocytic lymphohistiocytosis associated with excessive cytokine activation, while (Jia et al. 2023) described vaccine-associated Sweet syndrome characterized by neutrophil-driven inflammation (Chung 2025; De Clercq 2006). Although such complications are uncommon, they underscore the importance of identifying antiviral agents that may also possess anti-inflammatory or cytoprotective properties. In the present study, vitexin and isovitexin demonstrated substantial protection of Vero cells from virus-induced cytopathic effects. While inflammatory biomarkers were not assessed, the documented antioxidant and immunomodulatory properties of these flavone C-glycosides may partly contribute to the observed cytoprotective activity and warrant further mechanistic investigation.

Flavonoids are known to interfere with multiple stages of the viral life cycle, including viral enzyme activity, entry processes, and replication machinery (Song 2024). Vitexin (apigenin-8-C-β-D-glucopyranoside) and isovitexin (apigenin-6-C-β-D-glucopyranoside) are C-glycosylated flavones differing in the position of the glucose moiety. Their C–C glycosidic bond confers greater metabolic stability compared with O-glycosides (Sood 2010). Both compounds possess multiple hydroxyl groups that contribute to antioxidant potential and possible interactions with viral proteins. Despite relatively limited oral bioavailability due to first-pass metabolism, vitexin has been reported to bind human serum albumin and distribute systemically (Šudomová and Hassan 2023), suggesting potential pharmacological relevance.

The present study demonstrated that vitexin and isovitexin exhibited notable in vitro antiviral activity against HAV, HSV-1, HSV-2, and CoxB4, with comparatively weaker effects against RVA. Vitexin showed particularly strong activity against CoxB4 and demonstrated favorable selectivity indices. The reduced activity against RVA may reflect differences in viral replication mechanisms, receptor usage, or intrinsic resistance patterns associated with rotavirus biology (Vijayan et al. 2004). In contrast, the crude leaf and flower extracts displayed relatively greater activity against RVA, suggesting the possible contribution of additional phytoconstituents or synergistic interactions.

Previous reports support the antiviral potential of vitexin. Fahmy et al. described significant activity of vitexin against HAV and HSV-1 (Vilhelmova-Ilieva et al. 2023), while de Sousa et al. reported activity against hepatitis B virus and herpes simplex viruses (Xu et al. 2022). Mechanistically, vitexin and isovitexin have been shown to inhibit viral enzymes such as neuraminidase and reverse transcriptase in certain viral models (Yousef et al. 2025), although the exact mechanisms underlying their activity against the viruses tested here remain to be elucidated.

Importantly, both compounds demonstrated low cytotoxicity toward Vero cells, resulting in selectivity index values within ranges generally considered acceptable for preliminary antiviral evaluation (Zakzouk et al. 2025). However, it should be noted that cytotoxicity and antiviral activity were assessed using MTT- and CPE-based assays, which measure host cell viability indirectly rather than direct viral replication. Therefore, these findings should be interpreted cautiously as preliminary indicators of antiviral potential, not definitive evidence of viral inhibition.

HPTLC analysis confirmed the presence of vitexin in *Jatropha integerrima* leaves, consistent with previous reports describing flavone C-glycosides within the *Jatropha*genus. The successful isolation and structural characterization of vitexin and isovitexin further support the phytochemical relevance of this species as a source of bioactive flavones. However, correlation between compound concentration and antiviral activity requires further quantitative and mechanistic investigation (Zekeya et al. 2022). The successful isolation and structural characterization of vitexin and isovitexin from*J. integerrima*leaves, based on NMR and HRESI-MS analyses (Zhang et al. 2023), further support the phytochemical relevance of this genus as a source of bioactive flavones. Nevertheless, correlation between compound concentration and antiviral activity requires additional quantitative and mechanistic evaluation. Although certain Jatropha species exhibited higher vitexin content than*Jatropha integerrima*, the present study focused specifically on compounds isolated from *J. integerrima*. Comparative biological evaluation of other species warrants further investigatio.

Molecular docking analyses suggested that vitexin and isovitexin may interact with multiple SARS-CoV-2–related targets, including Mpro, spike glycoprotein, nucleocapsid phosphoprotein, the ACE2 receptor–RBD complex, and nsp10. While these computational findings indicate potential multi-target binding at the structural level, they remain predictive in nature and do not substitute for functional validation in viral infection models. Collectively, the present results provide preliminary computational support for further investigation of vitexin and isovitexin as candidate antiviral agents, while acknowledging the exploratory scope of both the in vitro and in silico analyses.

### Limitations of the study

Several limitations of this study should be acknowledged: Reference antiviral selection: Acyclovir was used as a reference drug; however, it primarily targets DNA viruses and may not serve as an optimal comparator for RNA viruses such as HAV and CoxB4. Indirect antiviral assessment: Antiviral activity was evaluated using MTT- and CPE-based assays, which measure host cell viability rather than direct viral replication. These assays may be influenced by cytotoxicity or metabolic effects, and therefore provide only indirect evidence of antiviral potential. Absence of direct viral quantification: Direct measurement of viral replication (e.g., plaque reduction assay or quantitative PCR) was not performed. Future studies should incorporate these methods to confirm the antiviral effects observed. SARS-CoV-2 evaluation: The activity against SARS-CoV-2 was assessed solely through in silico molecular docking, providing predictive but not experimentally validated evidence. Single cell line limitation: All experiments were conducted in Vero cells, which may not fully recapitulate human infection models. Additional studies in human-relevant cell lines and *in vivo* models are needed to validate these findings. Conclusion of limitations: Taken together, the results should be interpreted as preliminary and hypothesis-generating, highlighting the need for further mechanistic and translational validation.

### Recommendations for future studies

Direct viral quantification: Future studies should include direct measurement of viral replication using plaque reduction assays and quantitative PCR (qPCR) for viral RNA or DNA.

Reference antiviral selection: Mechanistically appropriate reference antivirals should be used, particularly for RNA viruses such as HAV and CoxB4, to provide more relevant comparative data. Experimental validation of in silico results: The predicted interactions of vitexin and isovitexin with SARS-CoV-2 proteins should be confirmed using cell-based infection models to validate computational findings. Mechanistic investigations: Studies examining viral entry, replication pathways, and host-response modulation would provide deeper insight into the modes of action of the tested flavonoids. Expanded experimental models: Evaluation in additional human-relevant cell lines and *in vivo* models will be important to confirm antiviral efficacy, pharmacokinetics, and safety. Combination therapy potential: Investigating possible synergistic effects between vitexin, isovitexin, and established antiviral agents could identify enhanced therapeutic strategies. Overall recommendation: Implementing these approaches will strengthen the translational relevance of the findings and provide a more comprehensive understanding of the antiviral potential and mechanisms of these compounds.

## Conclusions

This study provides evidence that vitexin and isovitexin isolated from *Jatropha integerrima* exhibit cytoprotective effects in vitro against a panel of human viruses, including HAV, HSV-1, HSV-2, and CoxB4, with moderate to strong selectivity in Vero cells. The crude leaf and flower extracts showed comparatively higher activity against RVA. Molecular docking analyses suggested favorable predicted binding interactions of vitexin and isovitexin with several SARS-CoV-2 proteins; however, these findings were computational and require experimental validation. Because antiviral activity was assessed using MTT-based cytoprotection assays, the results reflect indirect evidence of antiviral potential. Overall, vitexin and isovitexin may represent promising candidates for further antiviral investigation. Their therapeutic potential against SARS-CoV-2 and other emerging viruses remains to be established through comprehensive mechanistic, translational, and *in vivo* studies.

## Supplementary Information

Below is the link to the electronic supplementary material.

ESM1 (XLSX 53.8 KB)

ESM2 (DOCX 1.30 MB)

## Data Availability

The authors confirm that the data supporting the study’s conclusions were included in the article. Supplementary materials contain raw data.

## Abbreviations

AC: Acetic acid–water (15:85 *v/v*)
AdV: Adenovirus
BAW: *n*-Butanol-Acetic acid–Water (4:1:5 *v/v*), upper layer
CC: Column chromatography
CC_50_: A half-cytotoxic concentration
CoxB4: Coxsackievirus B4
CPE: Cytopathogenic effect
DMEM: Dulbecco’s Modified Eagle Medium
HAV: Hepatitis A virus
HPTLC: High-performance thin-layer chromatography
HSV-1: Herpes simplex virus type 1
HSV-2: Herpes simplex virus type 2
IC_50_: A half inhibitory concentration
MNTC: The maximum nontoxic concentration
MTT assay: MTT [3-(4,5-Dimethylthiazol-2-yl)-2,5-Diphenyltetrazolium Bromide] colorimetric assay
PBS: Phosphate-buffered saline
RVA: Rotavirus A
SI: Selectivity index
TLC: Thin-layer chromatography
SARS‑CoV‑2: Severe acute respiratory syndrome coronavirus 2
