# Supplementary material for: Broad-spectrum antiviral potential of vitexin and isovitexin from Jatropha integerrima: in vitro cytoprotective effects and in silico insights
Source: Naunyn Schmiedebergs Arch Pharmacol. 2026 Apr 2;399(9):13851–71. doi: 10.1007/s00210-026-05186-z (PMC13357401; doi:10.1007/s00210-026-05186-z)
Supplement: Supplementary file 2 — (DOCX 1.30 MB) [file 210_2026_5186_MOESM2_ESM.docx]

**Supplementary Materials**

**Broad-Spectrum Antiviral Potential of Vitexin and Isovitexin from *Jatropha integerrima:* *In Vitro* Cytoprotective Effects and *In Silico* Insights**

Hala Sh. Mohammed^1^, Shimaa M. Khalifa^1^, Eman FS Taha^2,*^, Amal H. Ahmed^1^, Ibrahim. H. Eissa^3^, Ahmed M. Metwaly^4^, Mohamed Marzouk^5^

^1^Department of Pharmacognosy and Medicinal Plants, Faculty of Pharmacy (Girls), Al Azhar University, Cairo 11754, Egypt

^2^Department of Health Radiation Research, National Centre for Radiation Research and Technology, Egyptian Atomic Energy Authority (EAEA), Cairo, Egypt

^3^Department of Pharmaceutical Medicinal Chemistry & Drug Design, Faculty of Pharmacy (Boys), Al-Azhar University, Cairo 11884, Egypt

^4^Department of Pharmacognosy & Medicinal Plants, Faculty of Pharmacy (Boys), Al-Azhar University, Cairo, 11884, Egypt

^5^Department of Tanning Materials and Leather Technology, Chemical Industries Research Institute, National Research Centre, 33 El-Bohouth St. (Former El-Tahrir St.), Dokki, Cairo 12622, Egypt

**Appendix**

| Item | Subject |
| --- | --- |
| Figure S 1 | **Negative HRESI-MS spectrum of vitexin** |
| Figure S2 | **^1^H NMR spectrum of vitexin in DMSO-*d_6_*** |
| Figure S3 | **^13^C NMR spectrum of vitexin in DMSO-*d_6_*** |
| Figure S4 | **Negative HRESI-MS spectrum of isovitexin** |
| Figure S5 | **^1^H NMR spectrum of isovitexin in DMSO-*d_6_*** |
| Figure S6 | **^13^C NMR spectrum of isovitexin in DMSO-*d_6_*** |
| Figure S7 | **Co-crystallized ligand docked into the active site of COVID-19 A: main protease; B: spike glycoprotein; C: nucleocapsid phosphoprotein; D: membrane glycoprotein; and E: Nsp10.** |
| Figure S8 | **Vitexin ligand docked into the active site of COVID-19 A: main protease; B: spike glycoprotein; C: nucleocapsid phosphoprotein; D: membrane glycoprotein; and E: Nsp10.** |
| Figure S9 | **Isovitexin ligand docked into the active site of COVID-19 A: main protease; B: spike glycoprotein; C: nucleocapsid phosphoprotein; D: membrane glycoprotein; and E: Nsp10.** |

**Fig S1. Negative HRESI-MS spectrum of vitexin**


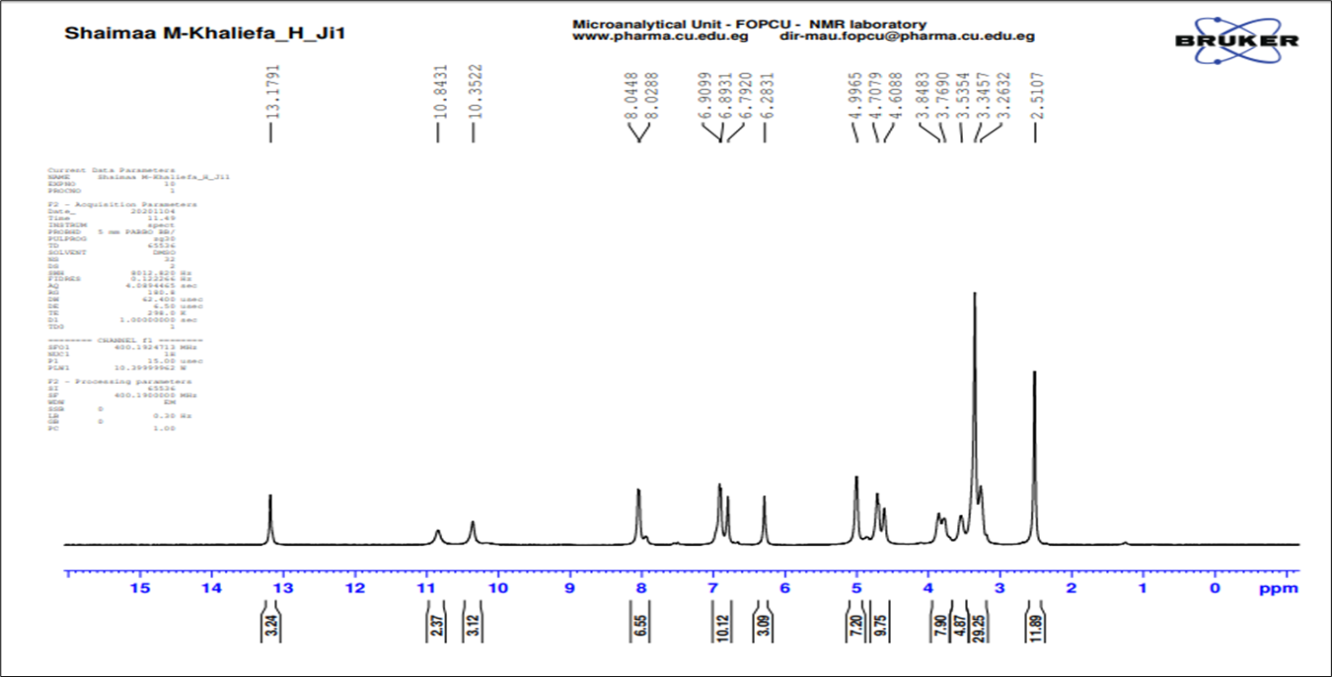

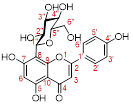


**Figure S2. ^1^H NMR spectrum of vitexin in DMSO-d_6_**

**Figure S3. ^13^C NMR spectrum of vitexin in DMSO-d_6_**


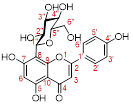


**Figure S4. Negative HRESI-MS spectrum of isovitexin**

**Figure S6. ^13^C NMR spectrum of isovitexinin DMSO-d_6_**


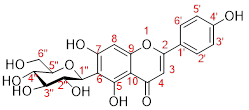


**Figure S5. ^1^H NMR spectrum of isovitexin in DMSO-d_6_**


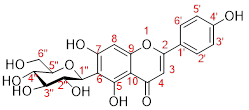


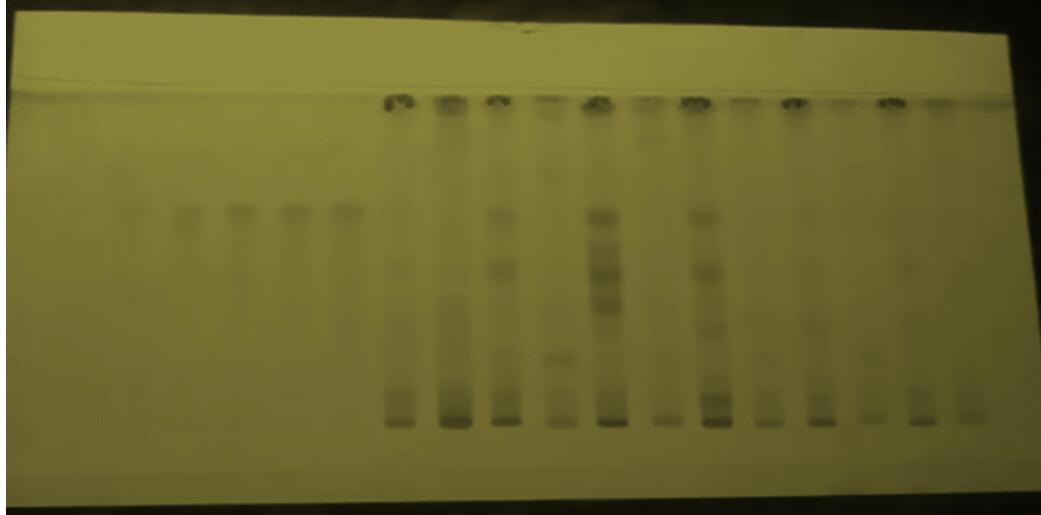


**Figure S7. The developed TLC chromatogram (original image)**
